# P-2052. From Challenge to Opportunity: Stewardship Strategies to Improve Blood Culture Utilization and Reduce Waste Amidst Global Shortage

**DOI:** 10.1093/ofid/ofaf695.2216

**Published:** 2026-01-11

**Authors:** William D Brown, Emily S Spivak, Valerie M Vaughn, Hannah Imlay, Chaorong Wu, Rachel Codden

**Affiliations:** University of Utah, Salt Lake City, Utah; University of Utah School of Medicine, Salt Lake City, Utah; University of Utah, Salt Lake City, Utah; University of Utah Health, Salt Lake City, UT; University of Utah, Salt Lake City, Utah; University of Utah, Salt Lake City, Utah

## Abstract

**Background:**

During the 2024 global blood culture (BCx) bottle shortage, the Antimicrobial Stewardship program at University of Utah Health created clinical guidance for the use of BCx implemented through education and a series of best-practice alerts (BPA). Here, we evaluate the interventions’ impact on BCx usage and hospital waste.

**Figure 1:**
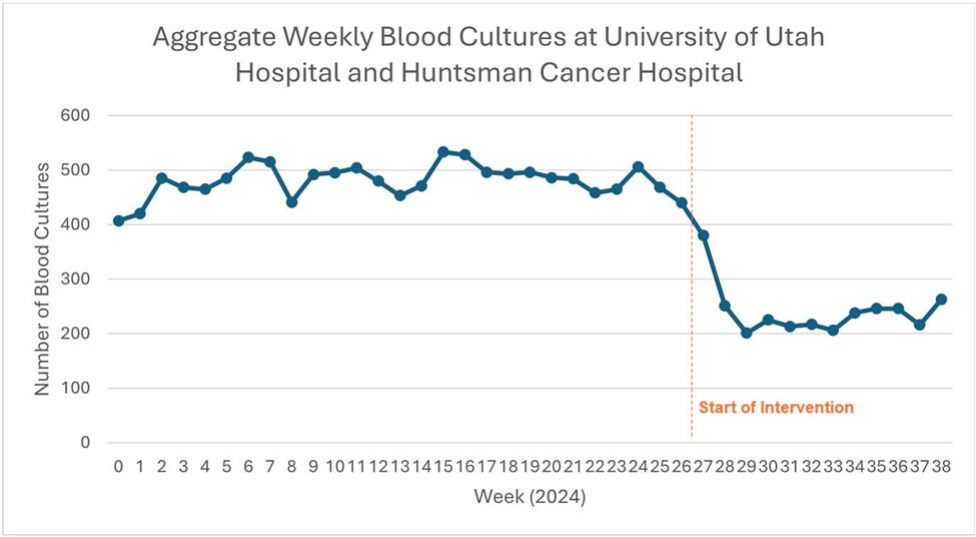
Number of blood cultures drawn per week across all units at University of Utah Hospital and Huntsman Cancer Hospital, including the Emergency Department. The vertical dashed line represents the start of the intervention. Average weekly BCx orders across all units decreased from 479.9 to 229.3, representing a 52.2% reduction.

**Figure 2:**
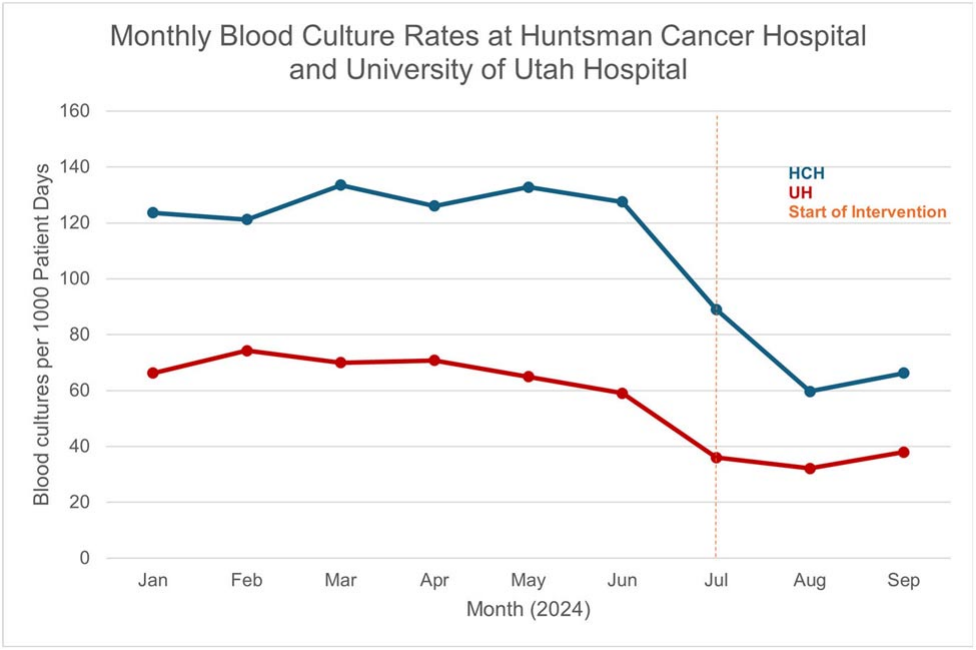
Monthly blood culture rates per 1000 patient days at two hospital sites, Huntsman Cancer Hospital (HCH) and University of Utah Hospital (UH) from January to September 2024. Emergency Department rates are not included due to lack of available census data. The vertical dashed line marks the beginning of the BCx stewardship intervention. Ordering rates decreased from 67.4 per 1,000 patient days to 35.0 per 1,000 patient days at University Hospital and from 127.4 per 1,000 patient days to 62.9 per 1,000 patient days at Huntsman Cancer Hospital.

**Methods:**

We rolled out escalating interventions over three weeks including: a) clinical guidance rendered via education, b) a BPA that was triggered by initial BCx orders to alert clinicians to the critical shortage and appropriate indications for BCx, and c) a BPA for repeat orders within 72 hours of an initial order including appropriate indications for follow up BCx. To assess the impact, we compared pre-intervention (01/01/24-07/11/24) vs. post-intervention (07/12/24-10/01/24) outcomes for our University (UH) and Cancer Hospital (HCH) including: a) unit- and hospital-level BCx ordering volumes adjusted per 1000 patient days, b) BCx positivity and contamination rates, and c) greenhouse gas emissions avoided.

**Figure 3:**
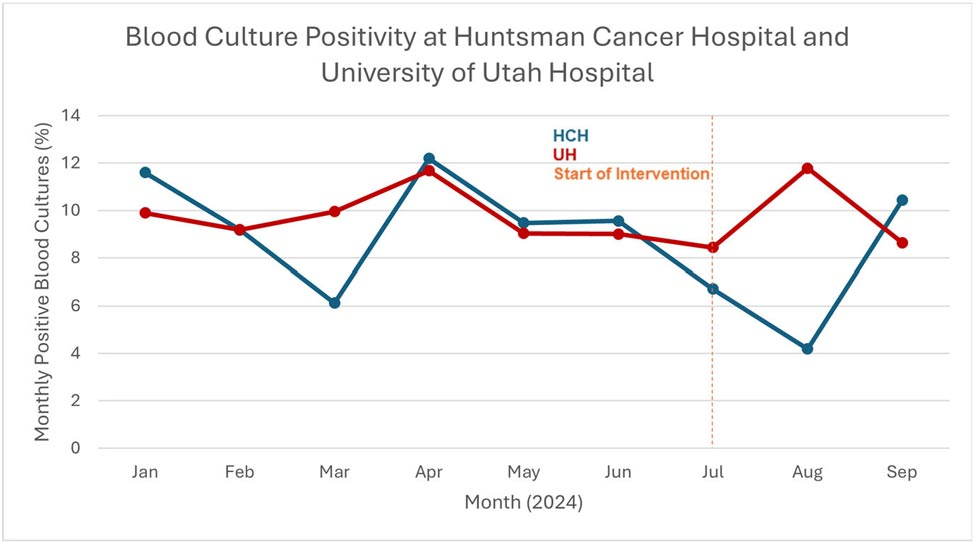
Montly blood culture positivity at University of Utah Hospital (UH) from January to September 2024. Emergency Department rates are not included. The vertical dashed line marks the beginning of the BCx stewardship intervention. The percentage of BCx that were positive did not change pre- vs. post-intervention (10.37% to 11.17%, p=0.22).

**Figure 4:**
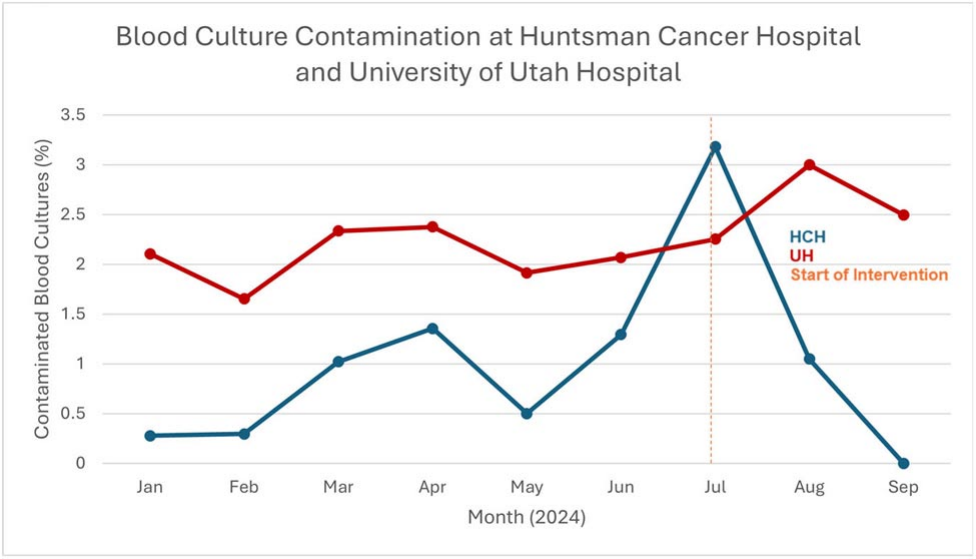
Monthly rates of blood culture contamination at University of Utah Hospital (UH) from January to September 2024. Emergency Department rates are not included. The vertical dashed line marks the beginning of the BCx stewardship intervention. The percentage of BCx that were contaminated did not change pre- vs. postintervention (2.17% vs 2.13%, p=0.89).

**Results:**

Of 17638 and 83585 patients admitted pre-intervention in HCH and UH, 12.75% and 6.74% had BCx drawn, respectively. Post-intervention, 6.29% (401/6387) and 3.5% (989/28,264) had BCx drawn at HCH and UH, respectively. During the intervention, average weekly BCx orders across UH and HCH decreased by 52.2% from 479.9 to 229.3 (Figure 1). BCx ordering rates at UH decreased by 48% and by 51% at HCH when adjusted per 1,000 patient days (Figure 2). The percentage of BCx that were positive (Figure 3) or contaminated (Figure 4) remained stable. A Chi-square test compared pre- and post-intervention BCx that were positive or contaminated. We estimate the reduction in BCx use saved 374kg of plastic, 0.007 metric tons of CO_2_, or emissions equivalent to burning 8 pounds of coal or driving 20 miles.

**Conclusion:**

Implementation of clinical support strategies and best practice advisories effectively improved BCx utilization and reduced waste in an academic center and a cancer hospital. Rates of positivity and contamination did not change, suggesting limited impact on patient outcomes. Given significantly reduced waste without changes in culture positivity, we recommend ongoing efforts towards effective and sustainable BCx stewardship.

**Disclosures:**

All Authors: No reported disclosures

